# Unified Convolutional Sparse Transformer for Disease Diagnosis, Monitoring, Drug Development, and Therapeutic Effect Prediction from EEG Raw Data

**DOI:** 10.3390/biology13040203

**Published:** 2024-03-22

**Authors:** Zhengda He, Linjie Chen, Jiaying Xu, Hao Lv, Rui-ning Zhou, Jianhua Hu, Yadong Chen, Yang Gao

**Affiliations:** 1 The Department of Computer Science and Technology, Nanjing University, Nanjing 210023, China; zhengda.he@cpu.edu.cn; 2 Laboratory of Molecular Design and Drug Discovery, China Pharmaceutical University, Nanjing 211198, China; chenlinjiecpu@outlook.com (L.C.); 3320051734@stu.cpu.edu.cn (J.X.);

**Keywords:** EEG analysis, sparse transformer, disease diagnosis, psychiatric drug discovery

## Abstract

**Simple Summary:**

Electroencephalograms provide valuable insights into brain activity with diverse medical utility that encompasses diagnosis, monitoring, drug discovery, and treatment evaluation. We propose an artificial intelligence model that is uniquely optimized to analyze electroencephalogram signals by directly processing raw data. This model captures intricate spatial and temporal patterns in electroencephalograms through dedicated components like spatial channel attention and sparse transformer encoding. Through being evaluated extensively, our model demonstrates high accuracy in detecting brain diseases and classifying psychotropic drugs. By automatically learning the representations from raw electroencephalogram data, it adapts well across diseases, subjects, and tasks. The model’s end-to-end learning aptitude and task versatility constitutes a robust and widely applicable automated electroencephalogram analytics solution. We believe it has the potential to significantly advance electroencephalogram-based diagnosis and personalized medicine.

**Abstract:**

Electroencephalogram (EEG) analysis plays an indispensable role across contemporary medical applications, which encompasses diagnosis, monitoring, drug discovery, and therapeutic assessment. This work puts forth an end-to-end deep learning framework that is uniquely tailored for versatile EEG analysis tasks by directly operating on raw waveform inputs. It aims to address the challenges of manual feature engineering and the neglect of spatial interrelationships in existing methodologies. Specifically, a spatial channel attention module is introduced to emphasize the critical inter-channel dependencies in EEG signals through channel statistics aggregation and multi-layer perceptron operations. Furthermore, a sparse transformer encoder is used to leverage selective sparse attention in order to efficiently process long EEG sequences while reducing computational complexity. Distilling convolutional layers further concatenates the temporal features and retains only the salient patterns. As it was rigorously evaluated on key EEG datasets, our model consistently accomplished a superior performance over the current approaches in detection and classification assignments. By accounting for both spatial and temporal relationships in an end-to-end paradigm, this work facilitates a versatile, automated EEG understanding across diseases, subjects, and objectives through a singular yet customizable architecture. Extensive empirical validation and further architectural refinement may promote broader clinical adoption prospects.

## 1. Introduction

Electroencephalogram (EEG) [1,2] is an electrophysiological technique that records brain electrical activity. EEGs measure weak the electrical signals produced by brain neurons through electrodes and converts them into graphical representations, thereby showcasing the temporal and frequency characteristics of the electrical activity. These waveforms reflect the activity and communication states of different brain regions, thus providing insights into brain functions and health status.

EEGs have been extensively utilized in brain function [1] research, clinical medical applications, and emerging applications, including brain–computer interfaces (BCIs) [2,3]. In terms of brain function research, EEGs have been used to explore fundamental mechanisms in neurobiology and brain plasticity, and studies have been conducted on cognitive processes such as memory, perception, decision making, and learning. In the realm of BCIs, EEGs aid disabled individuals by controlling external devices.

Medically, EEGs are used in disease diagnosis, monitoring, drug development, therapeutic effect evaluation, and prognosis prediction. For instance, EEGs are employed for diagnosing neuropsychiatric diseases like depression [4], schizophrenia, and attention deficit hyperactivity disorder (ADHD) [5]. Through EEG data analysis, physicians can observe specific patterns in the brain electrical activity of patients exhibiting motor disorder symptoms, thereby aiding in the early diagnosis of Parkinson’s disease. EEG monitors epileptic patients’ brain activity [6], where it tracks the frequency, intensity, and duration of epileptic seizures, thereby predicting seizures and locating their origin. By analyzing EEG data, doctors can monitor sleep apnea events in patients with sleep disorders [7]. Pharmaco-electroencephalography (Pharmaco-EEG) is a scientific method used to study the effects of drugs on brain electrical activity. Pharmaco-EEG is invaluable during drug development phases as it facilitates early drug screening to identify compounds with potential therapeutic effects. By integrating EEG biomarkers with neuropharmacology, Pharmaco-EEG offers a robust tool for studying the neural mechanisms of drugs, clinical efficacy, safety, and potential side effects. Pharmaco-EEG evaluates the effects of drugs in clinical treatments, including antidepressants, antiepileptic drugs, and sedatives. Monitoring EEG changes quantifies the improvement of drug symptoms. For instance, changes in Theta and Alpha waves are commonly used to assess the effects of antidepressant treatments. Moreover, certain EEG parameters, like the ratio of Delta and Beta waves, have been proven effective in predicting patient outcomes.

Traditional EEG analysis techniques encompass time-domain, frequency-domain, and spatial-domain analyses. Time-domain analysis, one of the most fundamental and widely used EEG analysis methods, is used to study EEG signal variations over time, which encompass the amplitude and time-domain feature calculations of the EEG signal. Frequency-domain analysis transforms the EEG signal from the time domain to the frequency domain using Fourier and continuous wavelet transforms, thereby revealing the associations between brain activity in different frequency ranges and specific neural processes. Spatial-domain analysis is used to examine the potential distribution of EEG signals at different scalp locations. Topographic maps [8] display the potential distribution of EEG signals on the scalp, thereby exploring the activity patterns and interactions of different brain regions. Source analysis aims to identify the active areas in the brain, which is crucial for locating brain lesions or studying brain functional connectivity. While traditional EEG analysis techniques offer high temporal resolution and cost-effectiveness, they have limitations, such as electrode position restrictions, sensitivity to noise, and the requirement for expert knowledge and high human resource costs.

In addressing the drawbacks of using traditional EEG analysis techniques, machine learning and deep learning technologies can analyze vast EEG data more precisely, thereby automatically recognizing patterns of drug effects and accelerating research progress.

Two core aspects of applying machine learning are feature extractor and classifier design. Traditional machine learning methods, including Support Vector Machines (SVM), Random Forests, and K-Nearest Neighbors, have been used to achieve some success but there are still the following challenges: dependence on manual feature engineering; difficulties in handling high-dimensional, multivariate EEG data; and potential inability to fully explore complex data relationships. Deep learning models [9] can automatically learn representative features without manual feature extractor design, and they can handle highly non-linear relationships. Convolutional neural networks (CNNs) and recurrent neural networks (RNNs) have shown excellent performance in feature learning from EEG data. However, CNNs and RNNs also face challenges: they are not adept at handling long-term information dependencies in long sequence data.

Recently, the transformer model, a deep learning architecture that has shown outstanding performance in natural language processing and computer vision, has also garnered attention in EEG data analysis. It has been employed for tasks such as emotion analysis, brain–computer interfaces, sleep analysis, and epilepsy detection. Comprising self-attention mechanisms, positional encoding, and multi-head attention, the transformer model excels in parallel processing and capturing long-distance dependencies, thus making it well suited for the temporal nature of EEG signals.

Most current research efforts first convert EEG signals into correlation matrices or interpolate them into images before processing with the transformer model. Such approaches might lead to a significant loss of signal information or could introduce noise. There is a pressing need to learn directly from the raw time-domain EEG signals. However, such studies typically employ standard transformer models, thus leading to two primary issues:

1. High computational cost: The self-attention mechanism requires calculations for every pair of time points, thereby posing challenges when dealing with long EEG time series;
2. Neglect of spatial correlations: EEG signals exhibit not only temporal dependencies, but also spatial correlations due to electrode placements. Standard transformer models primarily focus on temporal dependencies, potentially overlooking spatial correlations.

Given these challenges, further research should concentrate on optimizing the transformer model structure.

Moreover, due to the high specificity and significant variations of EEG data across different medical tasks, diseases, and patients, the current research is mostly confined to method and model design for single disease and medical tasks. This limitation significantly hampers the broader applicability of the technology. Developing a universal EEG analysis framework that can span across diseases, patients, and medical tasks is crucial yet challenging. Empirical studies on this universal framework, especially for key diseases like Major Depressive Disorder (MDD) and epilepsy, are essential.

**Figure 1:**
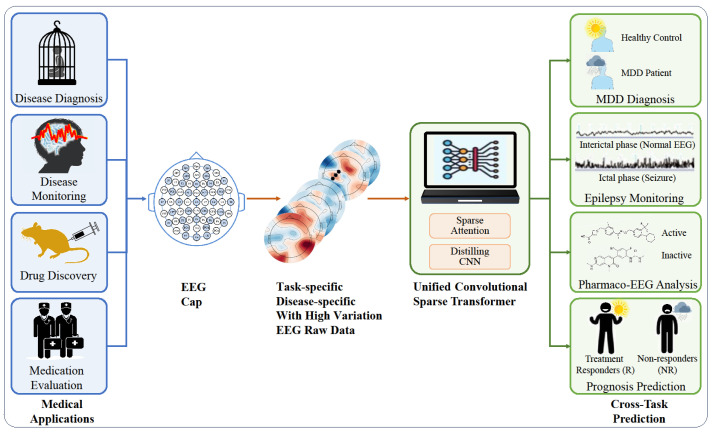
Unified convolutional sparse transformer for disease diagnosis, monitoring, drug development, and therapeutic effect prediction from EEG raw data.

To address the aforementioned issues, we propose the Convolutional Sparse Transformer, a universal EEG analysis framework. This framework can directly learn and extract features from raw EEG data. We introduced a spatial channel attention module to incorporate EEG spatial correlations. We also designed a sparse transformer to optimize the self-attention mechanism, thereby reducing computational costs and enhancing accuracy. To reduce the time dimension and select essential features, we employed a distillation convolutional layer. Owing to its intricate design and ability to learn from raw data, our method offers high performance and versatility. It is a universal framework that is adaptable to the specificity of different diseases and individual variations among patients, and it is suitable for cross-task scenarios like diagnosis, monitoring, drug discovery, and therapeutic effect evaluation, as shown in Figure 1. We conducted empirical studies on MDD and epilepsy, and we demonstrated the high performance and applicability of our framework across diseases, patients, and medical tasks. This paper is an expanded journal version of our previous work [10]. In this paper, we focused on proposing and analyzing the generalizability and cross-disease and cross-task capabilities of our model, as well as supplemented relevant new experimental results.

Our contributions are as follows:

1. We introduced a spatial channel attention module to capture the spatial dependencies of the EEG data;
2. We designed a sparse transformer and achieved an efficient temporal attention mechanism that can learn directly from raw EEG data;
3. Through the distillation convolutional layer, we successfully reduced the model’s time dimension while retaining key features, thereby enhancing the model’s computational efficiency and performance;
4. Our framework offers high versatility and it adapts to the specificity of different diseases and individual variations among patients, and it can be employed for various medical tasks, thereby filling a gap in the existing research;
5. Empirical studies on key diseases like MDD and epilepsy provide robust experimental evidence for the further application of this technology.

## 2. Related Work

An increasing number of studies have explored the utilization of advanced algorithms such as deep learning for the analysis of EEG data, thus aiming to achieve more accurate disease diagnosis, monitoring, and drug response analysis. This section provides an overview of the latest research advancements in this domain by focusing on critical medical conditions. Specifically, we review works related to the diagnosis of MDD using EEG, seizure monitoring, Pharmaco-EEG analysis, as well as drug response and prognosis prediction for MDD.

### 2.1. Deep Learning Models for the EEG-Based Diagnosis of Major Depressive Disorder

Recent advancements in deep learning have ushered in innovative EEG-based models for diagnosing Major Depressive Disorder. Mumtaz and Qayyum [11] achieved a remarkable 98.32% accuracy using a 1D convolutional neural network (1D-CNN), whereby they emphasized the potential of convolutional architectures. Wan et al.’s HybridEEGNet [12], while adept at capturing regional EEG features, reported a modest 79.08% accuracy, thus suggesting room for improvement. Acharya et al. [13,14] highlighted the significance of the right hemisphere in MDD diagnosis, but their model’s depth might raise overfitting concerns. Saeedi et al. [15] proposed a deep learning framework that utilizes 1D/2D CNN and CNN-LSTM architectures based on EEG connectivity. Another work by Saeedi et al. [16] introduced transformers. These studies, while pioneers, underscore the need for a more efficient and universally applicable model, thus paving the way for our Convolutional Sparse Transformer approach.

### 2.2. Deep Learning Models for EEG-Based Seizure Monitoring

Recent research has explored various deep learning architectures for seizure detection using EEG data. O’Shea et al. employed a fully convolutional architecture and achieved an AUC of 98.5% [17]. Frassineti et al. introduced a hybrid system that combines stationary wavelet transform with CNN, termed HybridEEGNet, thereby achieving an AUC of 81% [18]. Although effective, the wavelet transform adds computational complexity. Nagarajan et al. presented a machine learning architecture optimized for ultra-edge devices and achieved a sensitivity of 87% [19]. Tanveer et al. utilized an ensemble of 2D CNN models and achieved an average AUC of 99.3% [20]. While these models demonstrate the efficacy of deep learning in seizure detection, they also reveal gaps in computational efficiency.

### 2.3. Pharmaco-EEG Analysis Models

Pharmaco-EEG has recently gained prominence as a valuable tool for understanding the impact of drugs on the central nervous system. Kalitin et al. proposed a convolutional neural network for EEG-mediated drug–target interaction (DTI) prediction [21]. Despite its promise, the model faces challenges in prediction accuracy and computational efficiency. Manor et al. employed fast Fourier transform for the analysis of EEG profiles and sleep patterns in rats, thereby providing valuable insights into anxiolytic-like effects but lacking a machine learning approach [22].

### 2.4. EEG-Based Prediction of the Drug Response and Prognosis in Major Depressive Disorder

Mumtaz et al. utilized wavelet transform features and logistic regression to predict the treatment outcome for SSRIs in MDD patients, thereby achieving an accuracy of 87.5% [23]. More recently, Saeedi et al. proposed using transformers, outperforming traditional architectures like CNN and LSTM in both diagnosis and drug response prediction with an accuracy of 97.14% [16].

## 3. Method

### 3.1. Model Architecture

**Figure 2:**
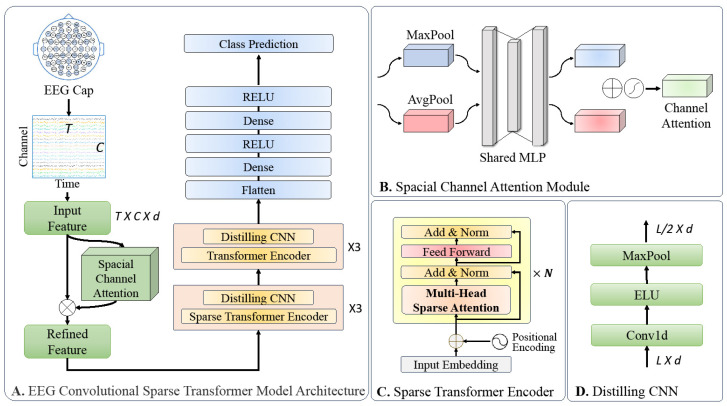
Architecture of the proposed EEG Convolutional Sparse Transformer.

The design motivation behind our Convolutional Sparse Transformer architecture stems from the need for a versatile, efficient, and domain-agnostic framework for EEG-based medical applications. The model was engineered to learn directly from raw EEG data, thus eliminating the necessity for expert-driven feature engineering. The architecture synergistically combines a spatial channel attention module, a sparse transformer encoder, a distilled convolutional layer, and a predictor module. These components were meticulously integrated to capture both spatial and temporal dependencies, reduce computational load, and adapt to various tasks, making the model a robust solution for disease diagnosis, monitoring, and prognosis prediction. As depicted in Figure 2, the architecture is composed of four key modules:

Spatial Channel Attention Module: This module is responsible for capturing the inter-channel correlations within the EEG signals, thereby enhancing the model’s ability to focus on relevant spatial features.

Sparse Transformer Encoder: This component employs a sparse attention mechanism to efficiently process the entire temporal domain of the EEG signals, thereby reducing computational complexity while maintaining performance.

Distilled Convolutional Layer: This layer serves to reduce the temporal dimensionality of the input features through maximum pooling, while also performing a distillation process to retain only the most salient features for subsequent processing.

Predictor Module: By utilizing the features extracted and refined by the preceding modules, this component performs the final task of signal classification.

### 3.2. EEG Input Signal Representation

In our model, the EEG signal is represented as a matrix X, where each row corresponds to a specific time frame and each column to an electrode channel. Let C denote the total number of electrode channels and T signify the total number of time frames sampled in a given epoch. Thus, X belongs to the set $R^{T\times C}$. Formally, the i-th row of X, denoted as $x_{i}$, is a vector in $R^{C}$ and can be expressed as $x_{i}=\left[x_{i,1},x_{i,2},\ldots ,x_{i,C}\right]$, where $x_{i,j}$ represents the amplitude of the j-th electrode channel at the i-th time frame.

### 3.3. Spatial Channel Attention Module

EEG signals manifest differently across various electrode channels, particularly during events like seizures. Moreover, the inter-channel correlations are unique and often overlooked in existing models, leading to feature interference and reduced predictive accuracy. To address this, we introduced a spatial channel attention module, which was designed to emphasize critical channel features and improve model performance. Inspired by the convolutional block attention module (CBAM) in computer vision [24], our module first applies global max pooling and average pooling to the EEG signal in the spatial channel dimension, thereby generating channel statistics. A multi-layer perceptron (MLP) then computes the channel attention, which is denoted as Mc⁡(X).

(1) $$\begin{array}{ll} Mc(X) & =\sigma (MLP(AvgPool(X))+MLP(MaxPool(X)))\\ & =\sigma \left(W_{1}\left(W_{0}\left(X_{avg}^{c}\right)\right)+W_{1}\left(W_{0}\left(X_{max}^{c}\right)\right)\right) \end{array},$$

Finally, the channel attention Mc⁡(X) is multiplied with the input EEG signal X to produce a feature-enhanced output. The inclusion of this module is crucial for capturing the spatial dependencies of EEG signals, particularly in the context of seizure events. By focusing on essential channel features, the module significantly enhances the model’s predictive accuracy.

### 3.4. Sparse Transformer Encoder Design

The development of sparse attention mechanisms in transformer models has become a focal point in recent research with several successful implementations [25,26,27]. Our work is particularly influenced by Zhou et al.’s informer model, which excels in long-sequence prediction tasks through a sparse encoder–decoder architecture [28]. However, our approach diverges by incorporating convolutional layers into a sparse transformer encoder, which is a novel combination aimed at efficient EEG data processing for disease detection and drug classification.

In the vanilla transformer architecture, each encoder layer updates its input embeddings using a global dense attention mechanism, which is mathematically represented as $A(Q,K,V)=softmax\left(\frac{QK^{T}}{\sqrt{d}}\right)V$. Here, $Q\in R^{L_{Q}\times d},K\in R^{L_{K}\times d}$, and $V\in R^{L_{V}\times d}$ denote the query, key, and value matrices for the input embeddings of the given layer, respectively. The dimensions $L_{Q},L_{K}$, and $L_{V}$ specify the number of rows in these matrices, while d represents the model’s feature dimensionality. The computational burden of this attention mechanism is $O\left(L_{Q}L_{K}\right)$, both in terms of time complexity and memory usage. This global dense attention mechanism serves as the baseline against which we compared our proposed sparse attention model.

Traditional vanilla transformers suffer from quadratic time complexity $O\left(L^{2}\right)$ [29], which limits their scalability for long sequences. Moreover, their global dense attention often introduces noise, thus affecting model performance. To mitigate these issues, our sparse transformer encoder employs selective global attention, thereby reducing time complexity to $O\left(L_{K}ln\left(L_{Q}\right)\right)$.

**Figure 3:**
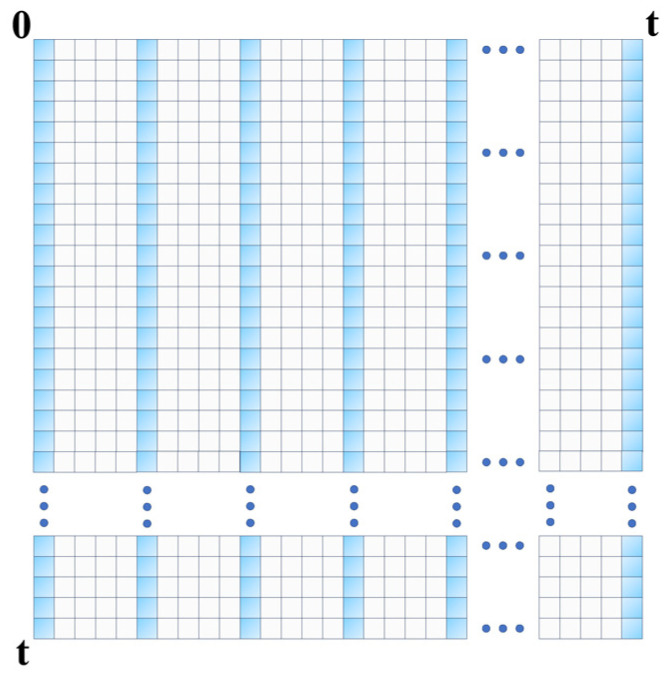
Attention mechanism in the sparse transformer encoder.

Specifically, as depicted in Figure 3, we selected Ln(L) terms at equidistant intervals from the input embedding for global attention. The attention for the i-th query is computed as follows:

(2) $$A\left(q_{i},K,V\right)=\sum_{j}\frac{k\left(q_{i},k_{j}\right)}{\sum_{l}k\left(q_{i},k_{l}\right)}v_{j},$$

where $k\left(q_{i},k_{j}\right)=exp\left(\frac{q_{i}k_{j}^{T}}{\sqrt{d}}\right)$.

For the remaining terms, we updated their attention using the mean value of the value matrix V:

(3) $$A\left(q_{i,\;\mathrm{other}\;},K,V\right)=Mean\left(v_{j}\right).$$

This design choice was justified by our experiments, which showed that selecting queries based on time intervals improves model performance compared to content-based selection.

### 3.5. Distilling Convolutional Layer

Incorporated after each encoder layer, the distilling convolutional layer serves dual functions. Firstly, it facilitates the fusion of temporally adjacent features, thereby leveraging the inherent strengths of convolutional neural networks in capturing local dependencies. Secondly, it employs a maximum pooling operation to reduce the temporal dimensionality of the input features. This not only streamlines the feature set, but also acts as a distillation process, thereby retaining only the most salient features for the subsequent layers. Mathematically, the feature transformation for each distilling convolutional layer can be expressed as follows:

(4) $$X^{j+1}=MaxPool\left(ELU\left(Conv1d\left(X^{j}\right)\right)\right),$$

where $X^{j}$ denotes the input feature matrix at the j-th layer and $X^{j+1}$ represents the updated feature matrix for the (j+1)-th layer. The exponential linear unit (ELU) activation function and the one-dimensional convolution operation (Conv1d) are applied sequentially before the maximum pooling operation (MaxPool).

## 4. Experiments

### 4.1. Experimental Tasks Design

We designed four experimental tasks:

(1) Disease Diagnosis Task: Classification of MDD patients and healthy individuals using the MDD dataset.
(2) Disease Monitoring Task: Classification between epileptic seizures and non-seizure states using the children epilepsy EEG dataset.
(3) Disease-Related Drug Discovery Task: Classification of psychiatric drug effects, mechanisms of action, and drug names using the Pharmaco-EEG dataset. The capability of the proposed model to perform these classifications can be used to evaluate the efficacy and mechanisms of new candidate drugs, as well as their potential indications. This enables the use of EEG signals as valuable biomarkers for drug screening and accelerates drug development.
(4) Disease Prognosis Prediction Task: In the MDD dataset, we predicted the potential responsiveness of MDD patients to Selective Serotonin Reuptake Inhibitors (SSRIs) as a means for the prognostic assessment of treatment outcomes.

### 4.2. Datasets

We conducted comparison experiments in the three datasets to validate the proposed model.

(1) Major Depressive Disorder Dataset [23,30]. This dataset was made publicly available by Mumtaz et al., and it includes resting-state EEG recordings from 34 MDD patients prior to medication. These recordings were captured under both eyes-closed and eyes-open conditions, each lasting for 5 min. Additionally, the dataset features EEG data from 30 age-matched healthy controls for comparative analyses. This dataset was used for the classification tasks between the MDD patients and healthy individuals. Furthermore, labels indicating the response to drug treatment were assigned based on the outcomes after 4 weeks of medication, which were evaluated by expert physicians using BDI and HADS scores. Out of the 34 MDD patients, 30 were labeled as either responders or non-responders to treatment. The dataset is suitable for both the EEG-based MDD diagnosis and prognostic prediction of drug responses in MDD patients.

(2) Helsinki children EEG dataset [31]. This dataset comprises multichannel EEG recordings from 79 neonates admitted to the neonatal intensive care unit at the University of Helsinki Hospital. The EEG signals were recorded at a sampling rate of 256 Hz using a 19-electrode cap configured according to the 10–20 international system. The median duration of these EEG recordings was 74 min. Three independent experts annotated the presence of seizures, with an average of 460 seizures annotated per expert. Based on consensus, 39 neonates had seizures and 22 were seizure free. The dataset includes EEG files in open-source EDF format and stands as the only publicly available dataset annotated by multiple experts for neonatal seizures, thereby serving as a reference for developing automated epilepsy monitoring algorithms.

(3) Pharmaco-EEG dataset [21]. The public dataset provided by Kalitin et al. contains intracranial EEG (i-EEG) signals recorded from rats after the administration of various psychotropic drugs. The i-EEG dataset comprises 16,500 samples from 5 rats for each of the 11 drugs at their maximum therapeutic doses. Each sample was 2 s long and contained 4-channel EEG data. The dataset includes 11 different psychotropic drugs, which are categorized into two types based on their effects: 7 are anticonvulsants and 4 are proconvulsants. These drugs can further be classified into five categories based on their mechanisms of action: (1) calcium channel blockers; (2) sodium channel blockers; (3) γ-aminobutyric acid (GABA) analogs; (4) GABA antagonists; and (5) cholinergic agents. The dataset is useful for classifying drug effects, mechanisms of action, and drug names based on EEG data, thereby facilitating EEG-based drug-target interaction predictions and drug screening.

### 4.3. Preparation of EEG Data

In the scope of our study, raw EEG signals were utilized, with the only modifications being filtering and segmentation. For the dataset associated with MDD, the signals were initially captured at a sampling frequency of 256 Hz. These signals were then subjected to a band-pass filter with cutoff frequencies set between 0.1 and 70 Hz. The 0.1–70 Hz range was selected to capture the main EEG frequency bands, including delta (0.5–4 Hz), theta (4–8 Hz), alpha (8–13 Hz), beta (13–30 Hz), and gamma (30–70 Hz). These frequency ranges have been extensively studied in the context of MDD and have been associated with various cognitive and emotional processes relevant to the disorder. By preserving this wide range of frequencies, we aim to retain the informative EEG components while attenuating low-frequency drifts and high-frequency noise that may contaminate the signals. Subsequently, 8 s segments were extracted from the filtered signals to form individual data points. The 8 s segment length provides a balance between the temporal resolution needed to detect dynamic changes in EEG patterns associated with depression, such as alterations in alpha asymmetry and the theta–beta ratio, and the computational efficiency of the model. In accordance with the existing literature on baseline methodologies, we allocated the data into training and test subsets using a 7:3 split ratio.

For the Helsinki dataset, the raw EEG signals were processed using a high-pass filter with a 0.5 Hz cutoff frequency to eliminate the slow baseline drifts and DC offsets that are common in this population. This cutoff frequency ensures that the relevant EEG activity, including seizure-related patterns, is retained while minimizing the influence of low-frequency artifacts. The data was then segmented into time windows of 4 s, 8 s, and 16 s, thus resulting in datasets of varying temporal lengths. Neonatal seizures exhibited variable durations and morphologies, and by using different segmentation lengths, we can assess the model’s robustness to these variations and identify the optimal temporal scale for seizure detection. Each segmented sample was characterized by $t^{*}f^{*}ch$ data points, where t denotes the duration of the time window in seconds, f signifies the sampling frequency, and ch indicates the number of electrode channels involved.

In the case of the Pharmaco-EEG dataset, segmentation was performed based on 2 s time windows. The Kalitin dataset is annotated in three distinct manners: (1) according to the drug name, (2) based on the drug’s mechanism of action, and (3) in terms of the drug’s efficacy. Consequently, the EEG signals can be sorted into 11, 5, and 2 categories based on these three criteria, respectively.

### 4.4. Configuration of the Hyperparameters for Experimental Models

Our study highlights the crucial role of hyperparameter search and optimization in developing high-performance deep learning models. The proposed Convolutional Sparse Transformer model involves multiple hyperparameters that collectively influence the model’s performance and generalization ability. We found a suitable hyperparameter configuration through a transformer expert knowledge-driven initialization and partial optimization using probabilistic hyperparameter optimization techniques.

Table 1 delineates the hyperparameter configurations employed for the proposed model across three distinct datasets. Each model architecture incorporates 3 layers of sparse attention and 3 layers of full attention, complemented by 5 convolutional and 5 max-pooling layers. The attention mechanism utilizes 8 heads, and the hidden layer dimensions are set at 128.

For the Pharmaco-EEG dataset, which involves a more complex multi-class classification task, we opted for a higher number of epochs, a smaller batch size, and a reduced learning rate. Conversely, for the binary classification tasks on the Helsinki and MDD datasets, fewer epochs were used along with a larger batch size and an elevated learning rate.

### 4.5. Baseline Methods

In the tasks of the MDD disease diagnosis and prognosis predictions for drug treatment, we selected baseline models from the literature [16], which included 1D CNN, LSTM, CNN-LSTM, and the standard transformer model. These models are representative of the current state-of-the-art in MDD diagnosis research when using EEG data, with the transformer model showing superior performance.

Our proposed Convolutional Sparse Transformer model introduces several architectural innovations that distinguish it from the baseline methods. A key feature of our model is the spatial channel attention module, which captures inter-channel correlations in EEG data. In contrast, baseline models such as 1D CNN, LSTM, and CNN-LSTM do not explicitly model the spatial relationships between channels. The 1D CNN baseline applies convolutional operations along the temporal dimension of EEG signals, thereby learning local temporal patterns. The LSTM and CNN-LSTM baselines capture temporal dependencies through recurrent connections and convolutional operations, respectively. While these models have demonstrated effectiveness in learning temporal patterns, they may not fully utilize the spatial information present in EEG data. Another significant architectural difference lies in the sparse transformer encoder, which efficiently processes long EEG sequences using a sparse attention mechanism. The sparse attention reduces the computational complexity from$\;O\left(n^{2}\right)$ to O(nLn⁡n), where n is the sequence length, thereby making it more scalable for handling lengthy EEG recordings. This is particularly advantageous compared to the standard transformer model used as a baseline, which has a quadratic complexity and may struggle with long sequences due to memory constraints.

For the disease monitoring task, specifically the epilepsy seizure monitoring, we drew upon models delineated in the literature [18,19] as our foundational models. The literature [18] has introduced two distinct models: a CNN configuration and an FCN variant. The former integrates convolutional layers with fully connected ones, whereas the latter, the FCN, substitutes the fully connected layers in the CNN with convolutional and pooling layers. Furthermore, the literature [19] has employed both the KNN, a staple in machine learning, and the ProtoNN model.

ProtoNN learns a sparse projection matrix to reduce the dimensionality of EEG data, while KNN is a non-parametric method that assigns labels based on the nearest neighbors in the feature space. The CNN and FCN baselines, as well as the ProtoNN and KNN baselines, have shown promising results in epilepsy seizure detection. Although these methods exhibit good performance, they may not capture complex spatial–temporal patterns as effectively as deep learning models like our Convolutional Sparse Transformer.

In the task related to disease-specific drug discovery, we selected the autoencoder model proposed in the literature [21] as the baseline model. The encoder of the autoencoder consists of convolutional layers and a pooling layer to extract features from drug-related EEG data. The classification of drugs via EEG is completed by passing the output of the encoder layer to a predictor composed of a fully connected neural network.

Our model employs a combination of convolutional layers and pooling operations in the feature extraction stage, thereby enabling it to learn the hierarchical representations of EEG data while reducing spatial dimensions. This allows for more efficient processing and increased scalability compared to the fully connected architectures used in the autoencoder baseline.

A potential limitation is the generalizability of the baseline methods to different EEG datasets or tasks. Some of these methods may have been originally proposed and evaluated on specific datasets with certain characteristics, such as the number of channels, sampling rate, or patient population. It is important to consider how well these methods adapt to variations in the data or task requirements. Our Convolutional Sparse Transformer model is designed to be flexible and adaptable to different EEG recognition scenarios, as we will demonstrate in our experiments, where it excels across multiple datasets and tasks.

### 4.6. Evaluation Metrics

In this study, we employed a comprehensive set of evaluation metrics to assess the performance of our proposed Convolutional Sparse Transformer model and compared it with the baseline methods across various EEG-based medical diagnostic tasks. The selected metrics include accuracy, precision, recall, F1-score, and ROC-AUC, each providing a unique perspective on the model’s performance.

Accuracy serves as an overall measure of the model’s correctness, and it is used to calculate the proportion of correctly classified samples. It is defined as follows: $Accuracy=\frac{TP+TN}{TP+TN+FP+FN}$, where TP, TN, FP, and FN represent true positives, true negatives, false positives, and false negatives, respectively. While accuracy offers a simple, intuitive understanding of performance, it can be misleading when the class distribution is imbalanced—a common occurrence in medical datasets.

Precision and recall are particularly relevant in medical diagnostic settings, where the costs of false positives (incorrectly identifying a healthy subject as diseased) and false negatives (incorrectly identifying a diseased subject as healthy) can be significant. Precision measures the proportion of true positive predictions among all positive predictions, which is defined as follows: $Precision=\frac{TP}{TP+FP}$. Recall, on the other hand, measures the proportion of true positive predictions among all actual positive samples, which is defined as follows: $Recall=\frac{TP}{TP+FN}$. These metrics provide insights into the model’s ability to minimize false positives and false negatives, respectively.

The F1-score, the harmonic mean of precision and recall, provides a balanced measure of the model’s performance, which is especially useful when dealing with imbalanced class distributions. It is calculated as follows: $F1-Score=2\times \frac{Precision\times Recall}{Precision+Recall}$.

ROC-AUC, the area under the receiver operating characteristic (ROC) curve, summarizes the model’s ability to discriminate between classes across different classification thresholds. The ROC curve plots the true positive rate (TPR) against the false positive rate (FPR) at various thresholds, where $TPR=\frac{TP}{TP+FN}$ and $FPR=\frac{FP}{FP+TN}$. A higher ROC-AUC value indicates a better model performance, with a value of 1 representing perfect classification and 0.5 indicating random guessing.

To provide a more nuanced understanding of our model’s performance, we also reported class-specific precision, recall, and F1-score values, thereby enabling insights into the model’s performance for each diagnostic class. Furthermore, confusion matrices were presented to visualize the model’s predictions and misclassifications across different classes, thus facilitating the identification of potential sources of errors.

Lastly, we discussed the clinical significance of the model’s performance in the context of EEG-based medical diagnostics by considering the practical implications of false positives and false negatives, as well as the potential impact on patient care and clinical decision making.

By employing this comprehensive set of evaluation metrics and analyses, we aim to provide a thorough and transparent assessment of our model’s performance, thus to better understand its strengths, limitations, and potential clinical utility in the context of EEG-based medical diagnostics.

## 5. Results

### 5.1. Results of the MDD Disease Diagnosis Experiment

In our study focusing on the diagnosis of Major Depressive Disorder, following the baseline source settings, we conducted ten experiments to evaluate the performance of our model, each time randomly dividing the dataset into training and testing sets with a 7:3 ratio. We employed a range of baseline models for comparison, including 1D CNN, LSTM, CNN-LSTM, and the standard transformer model, as cited from the existing literature [16].

The results, as tabulated in Table 2, clearly demonstrate the exceptional performance of our proposed model. These metrics not only outperformed all the baseline models, but also indicated a substantial improvement over the best-performing baseline, the transformer model. The improvements in ROC-AUC and F1-score were particularly noteworthy, thereby suggesting that our model is highly effective in both distinguishing between classes and achieving a balance between precision and recall. This validates the robustness and efficacy of our model in diagnosing MDD via EEG data.

The improved performance of our proposed Convolutional Sparse Transformer model, as demonstrated in Table 2, has the potential to significantly impact MDD diagnosis in clinical settings. Based on our dataset and experimental results, we estimated that the false negative rate in diagnosing MDD could be reduced from approximately 5.5% using the LSTM method to 0.3% using our method. This means that only 3 in 1000 MDD patients would be misdiagnosed as healthy, compared to 55 in 1000 using the LSTM method. Similarly, the false positive rate could be reduced from 3.5% to 0.6%, thus implying that 6 in 1000 healthy individuals would be misclassified as having MDD compared to the 35 in 1000 achieved using the LSTM method. These estimations highlight the potential of our method to enhance the accuracy and reliability of MDD diagnosis, thereby leading to earlier detection, more targeted interventions, and improved patient outcomes. Our model’s improved performance in MDD diagnosis has significant clinical implications.

**Figure 4:**
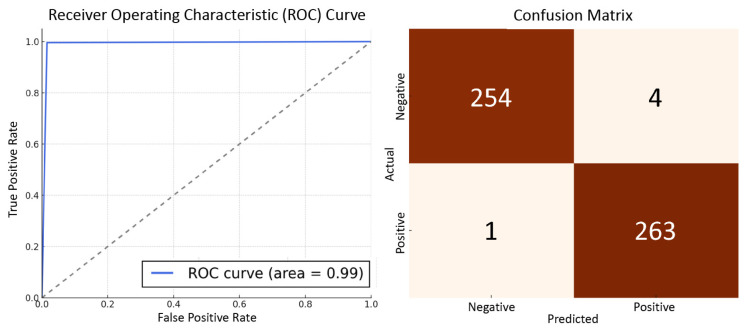
Performance evaluation of the Unified Convolutional Sparse Transformer in MDD diagnosis.

The left subplot of Figure 4 illustrates the ROC curve for the Unified Convolutional Sparse Transformer model on the MDD disease diagnosis test set, which highlights its discriminative power. On the right, the confusion matrix provides a detailed breakdown of the model’s true and false predictions.

### 5.2. Results of the Epilepsy Disease Monitoring Task

In our epilepsy monitoring experiments, we employed a 10-fold cross-validation strategy to assess the performance of our proposed model. The evaluation metrics are tabulated in Table 3. Across varying time window lengths of 4 s, 8 s, and 16 s, our model consistently outperformed the baseline models in all of the five key metrics: accuracy, precision, recall, ROC-AUC, and F1-score.

For instance, the highest accuracy among the baseline models for the 4 s window was 0.84 (FCN), which was markedly lower than our model’s performance. Similarly, our model’s ROC-AUC score of 0.976 is outstanding given that the highest among the baseline models with a reported ROC-AUC (as well as the FCN) was 0.79.

These results affirmed the robustness and efficacy of our model in monitoring epilepsy through EEG data, especially when employing a 4 s time window for analysis. With its ability to accurately identify seizure events from raw EEG signals, our model can assist clinicians in making prompt diagnostic decisions and initiating appropriate interventions.

While the CNN and FCN models are effective in learning local features, they may struggle to capture long-range dependencies due to their limited receptive fields. In contrast, our model’s sparse transformer encoder, coupled with the spatial channel attention mechanism, enables the efficient modeling of both local and global contexts in EEG data. On the other hand, the CNN and FCN models may be prone to overfitting, especially when dealing with limited training data. The performance of the KNN and ProtoNN models may be sensitive to the choice of distance metric and the quality of the extracted features. Our model addresses these limitations through its attention mechanism, which adaptively focuses on the most relevant features and prevents overfitting.

### 5.3. Results of the Psychotropic Drug Classification Experiment (Drug Discovery Task)

**Figure 5:**
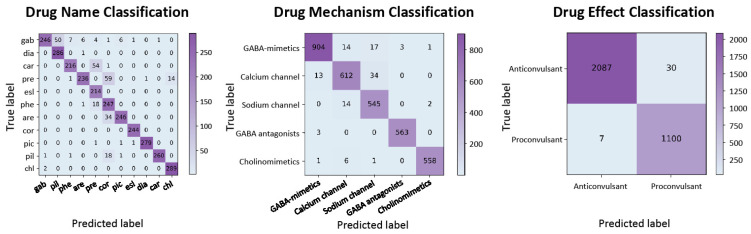
Confusion matrix for psychotropic drug classification.

In the context of drug discovery, we conducted experiments to classify psychotropic drugs based on their effects, mechanisms of action, and names using the Pharmaco-EEG dataset. The evaluation was carried out through five-fold cross-validation, and the results are tabulated in Table 4. Additionally, the confusion matrix is illustrated in Figure 5.

Our model demonstrates a marked improvement over the baseline autoencoder model across all evaluation metrics. These results underscore the robustness and efficacy of our model in classifying psychotropic drugs based on EEG data, thereby facilitating more accurate drug–target interaction predictions and expediting the drug development process.

The architectural differences between our model and the autoencoder contribute to its superior performance in capturing the complex spatio-temporal patterns in EEG signals associated with different psychotropic drugs. While the autoencoder is effective in learning the compressed representations of EEG data, it may struggle to capture long-range dependencies and temporal dynamics due to its fully connected architecture. Moreover, the performance of the autoencoder may be sensitive to the choice of hyperparameters, such as the number of layers and the dimensionality of the latent space.

### 5.4. Results of the Prognostic Prediction of the Drug Responses Experiment

In our study, we aimed to predict the responsiveness of MDD patients to Selective Serotonin Reuptake Inhibitors (SSRIs) using EEG data. Following the baseline source settings, we conducted ten experiments to evaluate the performance of our model, each time randomly dividing the dataset into training and testing sets with a 7:3 ratio. The evaluation metrics are summarized in Table 5. Our model demonstrated superior performance across all evaluation metrics, including in accuracy, precision, recall, ROC-AUC, and F1-score, when compared to the state-of-the-art baseline models.

Specifically, our model achieved an accuracy of 0.982±0.017 and an ROC-AUC of 0.994±0.005. These results were notably higher than the best-performing baseline model, the transformer. Moreover, the F1-score of our model stands at 0.985±0.014, which is a balanced measure of precision and recall, thus further confirming the robustness of our approach. These results underscore the efficacy of our model in predicting the treatment outcomes for MDD patients, making it a valuable tool for personalized medicine in mental health care.

Our model’s high performance in the prognostic prediction of drug responses experiment demonstrates its potential clinical utility. By accurately identifying patients who are likely to respond to SSRIs based on their EEG data, our model can assist clinicians in making informed treatment decisions, thus potentially reducing the trial-and-error process and improving patient outcomes.

### 5.5. Ablation Experiments

We chose to conduct ablation experiments on the mechanism of action classification task of epilepsy-related drugs, and the following three factors were considered in this study: 1. spatial channel attention, 2. transformer sparse attention, and 3. distillation convolution. The results of the experiment are shown in Table 6. It can be seen that the three improvement strategies for the transformer proposed in this study are effective. The distillation convolution operation is essential for the transformer to extract brain wave signal features, thus indicating that our proposed method of combining convolution and a transformer is effective for EEG raw data analysis.

## 6. Discussion

### 6.1. Hyperparameter Sensitivity Analysis

To systematically assess the impact of these hyperparameters, we conducted a hyperparameter sensitivity analysis on the 4 s dataset of the epilepsy disease monitoring task (Table 7). By varying the values of certain hyperparameters within reasonable ranges while keeping certain parameters fixed, we observed that the model’s performance was more sensitive to changes in some hyperparameters, such as the number of sparse-attention layers and CNN layers. This highlights the necessity of carefully tuning these hyperparameters to achieve optimal performance. By systematically exploring the hyperparameter space and finding the optimal configuration, we were able to fully leverage the potential of the Convolutional Sparse Transformer architecture and achieve a state-of-the-art EEG recognition performance.

### 6.2. Future Improvements to the Model Framework

The proposed Convolutional Sparse Transformer framework demonstrates its effectiveness in learning directly from raw EEG signals for various clinical tasks, such as disease diagnosis, monitoring, and prognosis prediction. The model’s ability to capture both spatial and temporal dependencies, along with its carefully designed components, enables it to extract informative features without the need for task-specific manual feature engineering. The uniformity mentioned in our paper refers to the consistent design of our deep learning model framework across different task domains.

In the current study, the unified framework was trained on each topic’s specific dataset and applied to the predictions within that particular domain. Consequently, the trained model’s weights and extracted features were distinct for each topic by adapting to the specific requirements of different tasks. This approach ensures that the model effectively learns task-specific patterns and achieves high performance in each domain.

However, an alternative approach worth exploring in the future is training a universal model for cross-topic clinical task detection. This model would have a shared feature extractor using the same weights across different topics, with multiple task-specific classifiers connected afterward. The sparse transformer proposed in this paper could serve as an effective foundation for such a feature extractor.

Training this universal model would involve a multi-task learning approach, where all of the topic tasks’ training sets are combined into a comprehensive dataset. Different task labels would be fed into their respective classifiers, and the loss functions would be combined to form an overall objective. This approach could potentially leverage shared information across tasks and improve the model’s generalization ability.

Nevertheless, developing a clinically universal model using multi-task learning requires careful design considerations. Balancing the training on datasets from different topics and achieving high-precision performance across various domains is challenging. Future research should focus on developing effective strategies for constructing comprehensive training sets and designing appropriate overall loss functions that optimize the model’s performance across multiple domains.

### 6.3. Enhancing the Interpretability of the Convolutional Sparse Transformer

While our proposed Convolutional Sparse Transformer model has demonstrated high performance in various EEG-based clinical tasks, the interpretability of its predictions is crucial for building trust and facilitating clinical acceptance. Interpretability enables clinicians and patients to understand how and why the model makes specific diagnostic decisions, thus promoting informed decision making and enhancing the overall reliability of the AI-driven system.

To enhance the interpretability of our model, several key strategies could be explored in future research. Firstly, feature importance techniques, such as attention visualization or gradient-based attribution methods, can be employed to highlight the specific EEG channels, time points, or frequency bands that contribute most significantly to a model’s predictions. By visualizing the attention weights or gradients, we can provide insights into which aspects of the EEG signal the model focuses on when making decisions. This information can help clinicians validate the model’s reasoning against their domain knowledge and identify potential biases or limitations.

Secondly, visualizing the learned representations at different layers of the model, such as the output of the spatial channel attention module or the sparse transformer encoder, can shed light on how the model progressively transforms and abstracts the raw EEG data into higher-level features relevant for the diagnostic task. Understanding these intermediate representations can provide valuable insights into the model’s internal workings and help identify potential sources of errors or uncertainties.

Thirdly, integrating domain-specific knowledge, such as known EEG biomarkers or clinical guidelines, into the model’s architecture or training process can make the model’s decision-making process more aligned with established medical knowledge and practices. This can be achieved by incorporating prior knowledge as constraints or as regularization terms in the model’s objective function, or by designing architectures that explicitly model domain-specific relationships or hierarchies. By leveraging domain knowledge, the model’s predictions become more interpretable and consistent with clinical expertise, thus increasing its acceptance and trustworthiness among healthcare professionals.

In conclusion, enhancing the interpretability of our Convolutional Sparse Transformer model is a critical direction for future research. However, achieving this goal requires close collaboration between us AI researchers, clinicians, and domain experts to ensure that the interpretability enhancements align with clinical needs and practices.

### 6.4. Applicability to Other Biomedical Signals

Although our current study primarily focuses on EEG signals and their applications in various clinical tasks, the proposed Convolutional Sparse Transformer model has the potential to be adapted for other types of biomedical signals. The core components of our model, particularly the sparse transformer with spatial channel attention, can be extended to capture the spatial and temporal dependencies present in signals such as electrocardiogram (ECG) and functional magnetic resonance imaging (fMRI) data.

In the case of ECG signals, the spatial channel attention module can be modified to capture the inter-lead dependencies, while the sparse transformer encoder can learn the temporal patterns associated with different cardiac events. For example, the spatial attention mechanism can be designed to focus on the most informative ECG leads for detecting specific cardiac abnormalities, such as arrhythmias or ischemia. The sparse transformer encoder can then learn the temporal characteristics of these abnormalities, thereby enabling the accurate diagnosis and monitoring of cardiovascular diseases.

Similarly, for fMRI data, the spatial channel attention module can be adapted to capture the functional connectivity between different brain regions, while the sparse transformer encoder can learn the temporal dynamics of the blood-oxygen-level-dependent (BOLD) signal. By leveraging the spatial and temporal information present in fMRI data, our model has the potential to contribute to the understanding of brain function and the identification of neurological disorders, such as Alzheimer’s disease or schizophrenia.

Furthermore, the Convolutional Sparse Transformer architecture can be extended to handle multi-modal biomedical signals, such as the combination of EEG and fMRI data, by incorporating additional attention mechanisms or fusion strategies. This has the potential to improve the accuracy and robustness of biomedical signal analysis.

### 6.5. Computational Environment and Cost

To evaluate the practicality of our proposed Convolutional Sparse Transformer model in real-world applications, we conducted a detailed analysis of its computational efficiency by focusing on runtime, memory requirements, and processing time. Our model was deployed on a representative hardware and software environment, including a workstation equipped with an Intel Core i9-11900K CPU@3.50GHz, 64 GB RAM, and an NVIDIA GeForce RTX 3090 GPU with 24 GB VRAM running Ubuntu 20.04 LTS (64 bit). The deep learning models were implemented using PyTorch 1.9.0 and trained using the CUDA 11.1 toolkit with cuDNN 8.0.5. The EEG data were loaded and processed using the MNE-Python 0.23 package.

In our experiments on the MDD diagnosis dataset, our model required an average training time of 12 min and an inference time of 1.39 s. The peak VRAM consumption was 3.2 GB, and the peak memory consumption was 9.2 GB. In our experiments on the Helsinki dataset for epilepsy monitoring, our model required an average training time of 75 min and an inference time of 5.1 s. The peak VRAM consumption was 3.9 GB, and the peak memory consumption was 22.6 GB. These computational requirements are within the capacity of modern workstations and GPUs, thus indicating that our model can be deployed on commonly available hardware. Compared to the baseline models, our approach achieved superior performance in terms of accuracy, F1-score, and other metrics while maintaining comparable computational costs. This demonstrated that our model provides state-of-the-art results without introducing significant computational overhead. We acknowledge that the scalability of our model to larger datasets or more complex architectures may require further optimization techniques, and future research should explore these optimizations.

Our analysis highlights the practical potential of the proposed Convolutional Sparse Transformer model in EEG-based medical diagnostic applications when considering its computational efficiency and deployment feasibility.

## 7. Conclusions

In this study, we proposed a novel Convolutional Sparse Transformer framework for learning directly from raw EEG signals in various clinical tasks, such as disease diagnosis, monitoring, and prognosis prediction. Our model, incorporating a spatial channel attention module, sparse transformer encoder, and distilled convolutional layer, has demonstrated a superior performance compared to existing methods by effectively capturing both the spatial and temporal dependencies in EEG data. The main contributions of our work lie in the development of a unified, end-to-end framework that eliminates the need for manual feature engineering and achieves state-of-the-art results across multiple EEG-based clinical applications. The proposed architecture has the potential to significantly improve diagnostic accuracy, enable earlier interventions, and facilitate personalized treatment plans, thereby ultimately enhancing patient care. However, we acknowledge the limitations of our current study, such as the need for further validation on larger, multi-center datasets to assess the generalizability of our findings. Future research directions include exploring multi-task learning approaches for developing a clinically universal model and investigating the transferability of our model to other EEG-based diagnostic tasks. In conclusion, our Convolutional Sparse Transformer framework showcases the power of deep learning in extracting meaningful insights from raw EEG signals. We believe that our work will inspire further research and collaboration between the machine learning and clinical communities.

## Figures and Tables

**Table 1:** Hyperparameter configurations across datasets.

Datasets	MDD	Helsinki	Pharmaco-EEG
Epoch	50	50	300
Batch Size	64	64	32
Learning Rate	$10^{-3}$	$10^{-3}$	$10^{-4}$
Sparse-Attention Layer	3	3	3
Full-Attention Layer	3	3	3
Attention Heads	8	8	8
$d_{\mathrm{model}}$	128	128	128
$d_{\mathrm{ff}}$	512	512	512
CNN Layers	5	5	5
Max-pooling Layers	5	5	5

**Table 2:** Performance comparison of the unified Convolutional Sparse Transformer model with the baseline methods [16] on the MDD disease diagnosis task.

Model	Accuracy	Precision	Recall	ROC-AUC	F1-Score
Transformer	0.972±0.007	0.954±0.014	0.994±0.003	0.971±0.008	0.974±0.007
CNN1D	0.964±0.013	0.963±0.016	0.968±0.015	0.964±0.013	0.965±0.012
LSTM	0.951±0.010	0.965±0.009	0.945±0.017	0.951±0.010	0.955±0.010
CNN-LSTM	0.970±0.009	0.973±0.014	0.970±0.010	0.970±0.009	0.971±0.008
**OURS**	0.996±0.003	0.994±0.005	0.997±0.004	1.000±0.000	0.996±0.003

The numbers in the table correspond to the mean ± standard deviation.

**Table 3:** Results of the epileptic seizure monitoring experiment (comparison with the baseline [18,19]).

Length	Model	Accuracy	Precision	Recall	ROC-AUC	F1-Score
4 s	CNN	0.83	0.41	0.39	0.77	0.40
	FCN	0.84	0.38	0.47	0.79	0.42
	ProtoNN	0.77	0.84	0.81	-	0.82
	KNN	0.78	0.8	0.76	-	0.78
	**OURS**	0.957±0.004	0.889±0.008	0.901±0.019	0.976±0.002	0.895±0.006
8 s	CNN	0.79	0.34	0.51	0.77	0.41
	FCN	0.82	0.39	0.63	0.81	0.48
	ProtoNN	0.77	0.85	0.83	-	0.84
	KNN	0.80	0.79	0.78	-	0.78
	**OURS**	0.953±0.004	0.872±0.013	0.888±0.040	0.967±0.006	0.879±0.021
16 s	CNN	0.85	0.73	0.37	0.75	0.49
	FCN	0.80	0.37	0.57	0.79	0.45
	ProtoNN	0.78	0.80	0.85	-	0.82
	KNN	0.78	0.77	0.75	-	0.76
	**OURS**	0.942±0.006	0.820±0.019	0.872±0.060	0.943±0.008	0.844±0.029

The numbers in the table correspond to the mean ± standard deviation.

**Table 4:** Results of the psychotropic drug classification experiment (comparison with the baseline methods [21]).

Model	Task Label	Classes	Accuracy	Precision	Recall	F1-Score
Autoencoder	Effect	2	0.814	0.789	0.803	0.795
	Mechanism	5	0.626	0.598	0.601	0.587
	Name	11	0.434	0.434	0.463	0.435
**OURS**	Effect	2	0.968±0.005	0.963±0.005	0.968±0.006	0.965±0.005
	Mechanism	5	0.964±0.003	0.960±0.004	0.964±0.003	0.962±0.004
	Name	11	0.832±0.007	0.834±0.007	0.835±0.007	0.832±0.008

The numbers in the table correspond to the mean ± standard deviation.

**Table 5:** Results of the prognostic prediction of the drug responses experiment (comparison with the baseline [16]).

Model	Accuracy	Precision	Recall	ROC-AUC	F1-Score
Transformer	0.970±0.007	0.973±0.005	0.978±0.013	0.968±0.006	0.975±0.006
CNN1D	0.936±0.020	0.936±0.021	0.959±0.018	0.930±0.022	0.947±0.016
LSTM	0.947±0.017	0.932±0.014	0.984±0.015	0.938±0.018	0.957±0.013
CNN-LSTM	0.944±0.015	0.933±0.012	0.978±0.017	0.936±0.015	0.955±0.012
**OURS**	0.982±0.017	1.000±0.000	0.971±0.027	0.994±0.005	0.985±0.014

The numbers in the table correspond to the mean ± standard deviation. The bold numbers indicate the highest scores.

**Table 6:** Results of the ablation experiments on the psychotropic drugs classification.

Spatial Channel Attention	Transformer Sparse Attention	Distillation	Accuracy	Precision	Recall	F1-Score
✓	✓	✓	0.964	0.960	0.964	0.962
×	✓	✓	0.939	0.935	0.940	0.937
✓	×	✓	0.947	0.942	0.948	0.944
✓	✓	×	0.753	0.749	0.749	0.749

The bold numbers indicate the highest scores.

**Table 7:** Hyperparameter sensitivity analysis results.

Hyperparameters	ROC-AUC	Precision	Recall	Accuracy
[2,2,3,3,512,128,8]	0.932 ± 0.013	0.860 ± 0.010	0.648 ± 0.029	0.945 ± 0.003
[3,3,5,5,512,128,8]	**0.976 ± 0.002**	**0.889 ± 0.008**	**0.901 ± 0.019**	0.957 ± 0.004
[4,4,7,7,512,128,8]	0.969 ± 0.006	0.876 ± 0.022	0.760 ± 0.029	**0.959 ± 0.003**
[3,3,5,5,512,32,8]	0.968 ± 0.002	0.787 ± 0.027	0.776 ± 0.030	0.949 ± 0.002
[3,3,5,5,512,64,8]	0.973 ± 0.003	0.818 ± 0.032	0.888 ± 0.028	0.954 ± 0.002
[3,3,5,5,512,128,4]	0.976 ± 0.002	0.826 ± 0.034	0.897 ± 0.006	0.956 ± 0.004
[3,3,5,5,256,128,8]	0.975 ± 0.002	0.834 ± 0.035	0.788 ± 0.017	0.956 ± 0.004
[2,2,3,3,512,128,8]	0.932 ± 0.013	0.860 ± 0.010	0.648 ± 0.029	0.945 ± 0.003

The content in the hyperparameters column represent the sparse-attention layers, the full-attention layers, the CNN layers, the max-pooling layers, the $d_{\mathrm{ff}}$, the $d_{\mathrm{model}}$, and the attention heads, respectively. The bold numbers indicate the highest scores.

## Data Availability

The Major Depressive Disorder Dataset is available at https://figshare.com/articles/dataset/EEG_Data_New/4244171 (accessed on 1 January 2024). The Pharmaco-EEG dataset is available at https://data.mendeley.com/datasets/gmkbhj28jh/1 (accessed on 1 January 2024).

## Abbreviations

The following abbreviations are used in this manuscript:

EEG	Electroencephalogram
Pharmaco-EEG	Pharmaco-electroencephalography
MDD	Major Depressive Disorder
